# Improved classification of rheumatoid arthritis with a score including anti-acetylated ornithine antibodies

**DOI:** 10.1038/s41598-020-73919-y

**Published:** 2020-11-06

**Authors:** Lorena Rodriguez-Martínez, Holger Bang, Cristina Regueiro, Laura Nuño, Ana Triguero-Martinez, Diana Peiteado, Ana M. Ortiz, Alejandro Villalba, Ana Martinez-Feito, Alejandro Balsa, Isidoro Gonzalez-Alvaro, Antonio Gonzalez

**Affiliations:** 1 Experimental and Observational Rheumatology and Rheumatology Unit, Instituto de Investigacion Sanitaria - Hospital Clínico Universitario de Santiago (IDIS), Travesia Choupana, sn., 15706 Santiago de Compostela, Spain; 2 Orgentec Diagnostika GmbH, Carl-Zeiss-Straße 49-51, 55129 Mainz, Germany; 3 Rheumatology Department, Instituto de Investigación Hospital Universitario La Paz (IDIPAZ), Paseo de la Castellana, 261, 28046 Madrid, Spain; 4 Rheumatology Department, Hospital Universitario de la Princesa, Instituto de Investigación Sanitaria La Princesa (IIS-lP), Calle de Diego de León, 62, 28006 Madrid, Spain; 5 Laboratorio Investigacion 10, Hospital Clinico Universitario de Santiago, Edificio de consultas, planta -2, Travesia de Choupana, sn, 15706 Santiago de Compostela, Spain

**Keywords:** Rheumatology, Rheumatic diseases, Rheumatoid arthritis

## Abstract

The presence of rheumatoid factor (RF) or anti-cyclic citrullinated peptide (anti-CCP) autoantibodies contributes to the current rheumatoid arthritis (RA) classification criteria. These criteria involve stratification on antibody levels, which limits reproducibility, and underperform in the RA patients without RF and anti-CCP. Here, we have explored if two anti-acetylated peptide antibodies (AAPA), anti-acetylated lysine (AcLys) and anti-acetylated ornithine (AcOrn), could improve the performance of the current criteria. The analysis was done in 1062 prospectively-followed early arthritis (EA) patients. The anti-AcOrn were more informative than the anti-AcLys, the conventional RA antibodies and the anti-carbamylated protein antibodies. The anti-AcOrn produced a classification that did not require antibody levels and showed improved specificity (77.6% *vs.* 72.6%, p = 0.003) and accuracy (79.0% *vs.* 75.8%, p = 0.002) over the current criteria. These improvements were obtained with a scoring system that values concordance between anti-AcOrn, RF and anti-CCP. No significant gain was obtained in sensitivity (80.2% *vs.* 78.8%, p = 0.25) or in improving the classification of the RA patients lacking RF and anti-CCP, although the anti-AcOrn ranked first among the analysed new antibodies. Therefore, the anti-AcOrn antibodies could contribute to the improvement of RA classification criteria by exploiting antibody concordance.

## Introduction

The patients with rheumatoid arthritis (RA) should be distinguished from other forms of arthritis for research and clinical management^1,2^. The prompt identification at the onset of arthritis is difficult to achieve because of the lack of discriminant symptoms or signs and the absence of diagnostic tests^1,3^. This limitation has been addressed through the elaboration of classification criteria by the American College of Rheumatology (ACR) and the European League Against Rheumatism, which primary focus is to identify homogeneous groups of patients for research^2,4^. The current criteria were developed in 2010 to avoid the delay associated with the previous criteria dating from 1987 to permit clinical trials early in the disease course. One of the novelties of the 2010 criteria has been a scoring system that gives a remarkable weight to the best-known RA specific autoantibodies, the rheumatoid factor (RF) and anti-cyclic citrullinated peptides (anti-CCP) antibodies^2^. Specifically, the patients showing high levels of any of the two antibodies receive 3 points, whereas the positive patients without high levels receive 2 points. These scores are a large fraction of the 6 points required for RA classification^2^. These and other changes in the 2010 EULAR/ACR criteria have achieved the intended objective of a much prompt classification^5,6^. However, there is still room for improvement^5–12^.

Two areas have been identified that could lead to improvements by incorporating new RA autoantibodies^5–7,13–15^. The most evident would be if the new antibody could cover the need of a biomarker for the 15–45% RA patients that lack RF and anti-CCP antibodies^7,13^. The accuracy of the current classification criteria for these “seronegative” patients is much lower than for the patients bearing RF or anti-CCP^5–8,11,12^. However, the new RA autoantibodies analysed so far have provided small gains in this area because of their concordance with RF and anti-CCP antibodies^16–18^. It is unclear if this characteristic also applies to the anti-acetylated peptide antibodies (AAPA)^18^. These antibodies are remarkable in comparison with other new RA autoantibodies^18–23^. Perhaps, they are only comparable in reproducibility and apparent diagnostic characteristics to the anti-carbamylated protein antibodies (anti-CarP)^24–26^, although the two are less sensitive for RA than RF and anti-CCP antibodies. The AAPA are assayed with a modification of the peptide from the mutated citrullinated vimentin (MCV) kit, in which citrulline is replaced by acetylated lysine (AcLys) or acetylated ornithine (AcOrn)^18,19^. The test shows low levels of cross-reactivity with anti-CCP and anti-CarP antibodies^18^. In spite of these promising clues, the potential value of the AAPA for RA classification has not been fully assessed. The available data shows discrepancies regarding their sensitivity for the “seronegative” patients. On one side, the report where they were first described observed a 13% sensitivity in the anti-CCP negative patients^18^. On the other, two meeting abstracts have reported > 40% sensitivity in the “seronegative” RA patients^20,21^. These latter results indicate the need to determine if the AAPA could significantly fill the need of a biomarker for the “seronegative” patients.

An alternative area of improvement of the 2010 EULAR/ACR classification criteria will aim to improve their specificity. Particularly, since the 2010 criteria show a loss of specificity relative to the 1987 criteria^5,6,10,12^. A loss that has been quantified at 4% across 12 studies^6^. The need for the highest specificity is taken very seriously because classification criteria are developed to obtain homogeneous groups of patients for research^3,5^. A way to obtain increased specificity has become possible very recently^14,15^. It exploits the concordance of autoantibodies to achieve high specificity, accuracy and reproducibility of the criteria. The concordance in antibody status, either positive or negative, is a well-known characteristic of RF and anti-CCP in the patients with RA. In other words, the fraction of antibody discordant subjects, subjects showing RF without anti-CCP or anti-CCP without RF, is much smaller in the RA patients than in the healthy controls and controls with other diseases^27–30^. This characteristic extends to the anti-CarP antibodies. In effect, the excess concordance of RF, anti-CCP and anti-CarP antibodies in the RA patients has been confirmed in a recent compilation of 12 studies^24^. All the studies showed the three antibodies were much more concordant in the RA patients than in the controls, either healthy controls, first degree relatives of RA patients, or disease controls^24^. This characteristic was exploited by us to replace the serological component of the ACR/EULAR 2010 classification criteria by a score based on the concordance of the anti-CarP, RF and anti-CCP autoantibodies. This score led to classify the patients with similar accuracy in our cohort^14^, and, independently in another large cohort of patients with EA^15^. A replication that highlights the advantage of a scoring system that does not require antibody concentrations, its reproducibility. In effect, antibody concentrations vary between laboratories and it has been shown to limit reproducibility of the RA classification criteria^10^.

Here, we have evaluated the two best-performing AAPA^18–20^. The first part of our analysis did not replicate the high sensitivity for seronegative patients previously reported by Studenic et al.^20,21^. However, the second part showed the anti-AcOrn antibodies led to the most accurate classification, significantly better than the 2010 ACR/EULAR classification criteria, with the concordance scoring system.

## Patients and methods

### Patients and samples

Patients included in the study comprised the 1062 EA patients used in a previous report^16^. They had been recruited in the PEARL (Princesa Early Arthritis Register Longitudinal) study^31^ at Hospital Universitario La Princesa (from July 2001 to December 2014) and at Hospital Universitario La Paz (from January 1993 to December 2013)^32^, both in Madrid. They presented 2 or more swollen joints for less than a year and were naïve for Disease-Modifying Anti-Rheumatic Drugs (DMARD) at the first visit. In addition, they had completed 2 years of follow-up and there was available serum from the baseline visit. At the end of the 2-year follow-up, these patients were classified according to the 1987 ACR classification criteria for RA^4^. This classification in RA and non-RA was taken as the gold standard for comparison. A choice based on the increase in sensitivity of the 1987 criteria at this time relative to the first visit. The EA clinic and the sample collections were approved by the La Paz University Hospital Ethics Committee and the Ethics Committee for Clinical Research of Hospital Universitario La Princesa (Ref. PI-518). The study was approved by the Autonomous Research Ethics Committee of Galicia (Ref. 2014/387 and 2017/514). All participants provided their written informed consent and all protocols and methods were conducted according to the relevant guidelines (Declaration of Helsinki, the Belmont Report and the Spanish Law of Biomedical Research no. 14/2007).

### Determination of autoantibodies

We measured IgG autoantibodies against 2 acetylated peptides derived from vimentin, one of them with acetylated lysine (anti-AcLys) and the other with acetylated ornithine (anti-AcOrn) at position 7 of the peptide. The ELISA was performed according to the Orgentec protocol described elsewhere^18^. No peptides without acetylated amino-acids were assayed given the low frequency of reactivity against them in previous studies. Two different lots of the peptides, plates and other reagents were provided by Orgentec (Orgentec Diagnostika GmbH, Germany), but they are not commercially available. The cut-off for positivity was defined as the 98^th^ percentile of antibody reactivity in the sera of 270 healthy controls from Hospital Clínico Universitario de Santiago (Santiago de Compostela, Spain). It corresponded to 64 U/ml for anti-AcLys and 55 U/mL for anti-AcOrn antibodies. The inter-assay coefficients of variability across all the ELISA plates were 3.8% and 3.3% for the anti-AcLys and anti-AcOrn antibodies using a control showing 159 and 152 U/mL, respectively. The status of the other autoantibodies (RF, anti-CCP and anti-CarP) was available from a previous study^16^. Precisely, the anti-CarP antibodies were determined using a homemade ELISA with in vitro carbamylated FCS, the IgM-RF was determined by nephelometry, and the anti-CCP antibodies by standardized ELISA. The particular anti-CCP kit was the anti-CCP2 Euro-Diagnostica Immunoscan RA (positive > 50 U/ml) for all patients in Hospital Universitario La Paz and until October 2010 in PEARL. Thereafter the QUANTA Lite CCP3 IgG and IgA assay of Inova Diagnostics (positive > 40 U/ml) was used in PEARL.

### Statistical analysis

Continuous patient variables were compared with the U of Mann–Whitney test or t-test according to their distribution, whereas dichotomous variables were compared with 2 × 2 contingency tables. Quantile normalization of the optical densities was used to correct for differences between the two anti-acetylated peptide ELISA lots. Most analyses considered only two statuses for each antibody, positive or negative, but other analyses considered three levels following the 2010 ACR/EULAR classification criteria^2^. These three levels were: negative, positive below 3 times the cut-off value for each antibody, and positive over 3 times the cut-off. The cut-offs for anti-CCP and RF antibodies were taken from the manufacturer and the 3 times cut-off value was separately calculated for each anti-CCP kit in each EAC. The cut-off for anti-AcLys and anti-AcOrn antibodies was calculated as described above whereas that of anti-CarP antibodies has been defined previously^26^. Concordance between antibody status was measured with the Goodman and Kruskal’s gamma coefficient (γ), (ranging from + 1 = perfect concordance to − 1 = complete discordance). In addition, the association between the antibodies and RA was assessed with logistic regression accounting for age, sex, the specific EA clinic, and the status of other autoantibodies. Other parameters of classification performance were sensitivity, specificity, positive predictive value (PPV), negative predictive value (NPV), positive likelihood ratio (LR+), negative likelihood ratio (LR−) and the area under (AUC) the receiver operating characteristic (ROC) curves. The sensitivity and specificity of different antibodies or antibody combinations were compared with the McNemar’s test for paired contingency tables. Also, we assessed the RA classification based on the concordance of the autoantibodies^14^. For the logistic regression analysis, only the main effects were ascertained. Moreover, the logistic regression fit to the data was assessed with the Nagelkerke R^2^, and the Akaike’s Information Criterion (AIC). The Nagelkerke R^2^ estimates the predictive power of the model as a proportional reduction in error variance. The AIC estimates the relative amount of information lost by any model. Therefore, the R^2^ increases with the predictive power of the model, whereas the AIC reaches lower values for the best models. Differences in AIC > 2 between any two models are meaningful, whereas differences > 10 are interpreted as rejecting the poorer model^33^. Finally, the impact of the different serological criteria on the overall classification (serological + non-serological criteria) was explored in the patients from PEARL, who featured all the required information. This exploration was undertaken in two ways. The first consisted of replacing the serological scores in the 2010 ACR/EULAR criteria. The second classified the patients with logistic regression that combined the non-serological and serological criteria applying cut-offs that were adjusted to obtain a constant sensitivity. The results of these classifications were expressed as specificity (true nonRA/observed nonRA patients), sensitivity (true RA/observed RA patients) and accuracy ((true nonRA + true RA)/all patients). The statistical tests were performed with R through the Jamovi application^34,35^ and Statistica version 7.0 (StatSoft, Tulsa, OK) except for the ROC analysis, which was done with SPSS version 15.0 (Chicago, USA). Area proportional Venn diagrams were produced with EulerAPE 3.0^36^.

## Results

### Prevalence of the anti-acetylated peptides antibodies in the EA patients

The 1062 patients with EA were divided at the end of the 2 years of follow-up into 49.9% with RA and 50.1% without RA. This latter group included patients with undifferentiated arthritis (20%) and other less common diseases that add up to the remaining 30.1%. The diseases in the latter group were spondyloarthritis, Sjögren syndrome, systemic lupus erythematosus, psoriatic arthritis, inflammatory bowel disease… The retrospective analysis showed that the RA and non-RA patients already differed in several features at the first visit: age, length of the symptoms, disease activity, presence of erosions and the prevalence of the autoantibodies (Table 1). In effect, the five antibodies, including the two AAPA, were very significantly associated with RA.

**Table 1:** Clinical and serological features of the EA patients.

	All = 1062	RA^a^ = 530	Non-RA = 532	P^c^
Women, n (%)^b^	818 (77.0)	420 (79.2)	398 (74.8)	0.09
Age, median (IQR ) years	52.0 (40.0–65.2)	54.0 (43.0–67.4)	49.2 (37.0–63.6)	2.5 × 10^–05^
Delay, median (IQR ) weeks	16.0 (8.0–28.1)	19.9 (11.0–30.7)	12.0 (6.0–26.4)	1.6 × 10^–10 #^
DAS28, median (IQR )	4.5 (3.4–5.8)	5.2 (3.9–6.3)	3.9 (2.9–4.9)	1.3 × 10^–34^
Erosions, n (%)^d^	93 (10.5)	77 (14.6)	16 (4.5)	1.9 × 10^–06^
Ever smoker, n (%)^d^	444 (45.1)	234 (46.0)	210 (44.2)	0.6
RF^+^, n (%)	444 (41.8)	358 (67.5)	86 (16.2)	1.3 × 10^–64^
anti-CCP^+^, n (%)	397 (37.4)	351 (66.2)	46 (8.6)	9.1 × 10^–84^
anti-CarP^+^, n (%)	291 (27.4)	222 (41.9)	69 (13.0)	4.4 × 10^–26^
anti-AcLys^+^, n (%)	142 (13.4)	111 (21.1)	31 (5.8)	2.9 × 10^–13^
anti-AcOrn^+^, n (%)	236 (22.2)	191 (36.1)	45 (8.5)	2.6 × 10^–27^
AAPA^+^, n (%)	259 (24.5)	209 (39.7)	50 (9.4)	2.2 × 10^–30^

^a^EA patients classified as RA or non-RA according to the 1987 ACR classification criteria at 2 years of follow-up.

^b^Features corresponding to the first visit are presented.

^c^p values correspond to the comparison of the RA and non-RA patients with the chi-squared or t-tests/U of Mann–Whitney test ^#^.

^d^Information was available for < 95% of the patients: ever smoker = 92.7%; erosions = 83.1%.

To complete the preceding analyses, the association between the presence of the AAPA and RA was assessed with logistic regression accounting for other antibodies and variables (Supplementary Table S1 online). This analysis showed that the anti-AcLys antibodies were no longer associated when the presence of anti-CCP and RF were considered. In contrast, the anti-AcOrn antibodies remained significantly associated when the other autoantibodies were considered, even when the anti-CarP antibodies were added. In the most complete analysis with four antibodies, the strength of the association with RA went in decreasing order from the anti-CCP antibodies (OR 10.0) to RF (OR 2.9), the anti-AcOrn antibodies (OR 1.6) and the anti-CarP antibodies (OR 1.6). The combination of the two AAPA was marginally more associated than the anti-AcOrn antibodies separately (Supplementary Table S1 online).

We also explored if the stratification of the AAPA by their levels could add discriminant value. Three levels were defined as in the ACR/EULAR 2010 classification criteria: negative and above or below 3 times the cut-off of the positive patients (Supplementary Table S2 online). Unlike the RF and anti-CCP antibodies, the high levels of the AAPA were not more associated with RA than the low levels. These results excluded the AAPA concentration strata from RA classification rules.

### Relations between the autoantibodies

**Figure 1:**
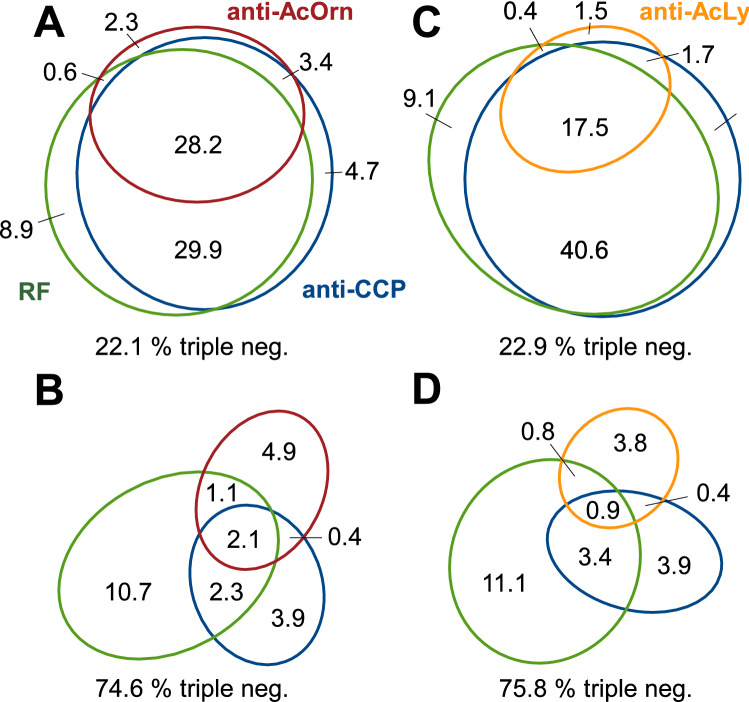
Relations of the AAPA with RF and the anti-CCP antibodies in the EA patients. The relations of the anti-AcOrn antibodies are shown in the (**A**) and (**B**) plots, whereas the anti-AcLys antibodies are shown in the (**C**) and (**D**) plots. The (**A**) and (**C**) plots represent the RA patients, whereas the (**B**) and (**D**) plots show the non-RA patients. The percentages are the fraction of the total.

The two AAPA were strongly concordant in status, both in the RA (γ = 0.89, p = 8.9 × 10^–70^) and non-RA patients (γ = 0.98, p = 1.5 × 10^–119^). As a consequence, their relations with RF and the anti-CCP antibodies were similar (Fig. 1). For the anti-AcOrn antibodies, the largest two strata of RA patients comprised the triple-positive and the anti-CCP/RF double-positive patients (Fig. 1A). The subset of patients positive only for the anti-AcOrn antibodies, which is of particular relevance to increase the sensitivity of RA classification, represented only 2.3% of the total (or 9.3% of the RF and anti-CCP seronegative RA patients). A completely different distribution was observed in the non-RA patients (Fig. 1B), where the largest subgroup was negative for the three antibodies. They were followed by the positive for unique antibodies: RF, anti-AcOrn or anti-CCP. A notably similar pattern of relations was observed with the anti-AcLys antibodies in the non-RA patients (Fig. 1D). In contrast, the distribution of the anti-AcLys antibodies in the RA patients did not resemble that of the anti-AcOrn antibodies: it was dominated by an enlarged subgroup of anti-CCP/RF double-positive patients followed by the triple-negative patients and a reduced subgroup of triple-positive patients (Fig. 1C). It was notable that the two critical subsets for improving RA classification were less frequent in the anti-AcLys than the anti-AcOrn antibodies (Fig. 1A,C): the patients positive only for the AAPA and the triple-positive patients.

### Diagnostic parameters of the AAPA

A variety of parameters assessing the AAPA contribution to RA classification were calculated (Table 2 and Supplementary Table S3 online). The fundamental parameters sensitivity and specificity were compared with the classical antibody combination: positive for “RF or anti-CCP” (Table 2). This analysis showed the AAPA were notably less sensitive than the classical antibody combination on the whole set of patients. In addition, the AAPA were positive in less than 11% of the anti-CCP^−^ or the anti-CCP^−^/RF^−^ patients. In contrast, the AAPA showed high specificity (Table 2). It was higher than the specificity of the “RF or anti-CCP” combination and it was preserved in the “seronegative” patients, both anti-CCP^−^ and anti-CCP^−^/RF^−^ patients. This high specificity suggested an improved classification could be obtained with an OR combination. The actual testing of the three- and four-antibody combinations showed very modest but significant increases in sensitivity (Table 2). The increases were of 1.4% with the anti-AcLys, 2.2% with the anti-AcOrn, and 2.5% with the combination of the two AAPA (p < 0.015 for all the comparisons with the “RF or anti-CCP” rule). These improvements were associated with a decrease in specificity that was of 3.7% with the anti-AcLys, 4.9% with the anti-AcOrn, and 5.4% with the AAPA combination (p < 2.5 × 10^–5^ for all the comparisons with the “RF or anti-CCP” reference). In other words, the benefit–cost ratio of the antibody combinations was 1:2.2 for the “RF or anti-CCP or anti-AcOrn antibodies” and the “RF or anti-CCP or anti-AcOrn or anti-AcLys antibodies” rules. This ratio meant that for each new correctly classified RA patient there will be 2.2 non-RA patients falsely classified as having RA. In turn, the benefit–cost ratio of the rule with the anti-AcLys antibodies was 1:2.6 and the recently reported with the anti-CarP antibodies was 1:3.7^16^. These values were not significantly different between them. As an alternative, we tested the value of combining the antibodies with the AND operator but this approach resulted in a notable decrease in the sensitivity, which is the parameter with the highest need for improvement (Supplementary Table S4 online). None of the other analysed parameters (Supplementary Table S3 online) showed a significant improvement with the AAPA.

**Table 2:** Sensitivity and specificity of the AAPA for the RA classification of the EA patients.

	anti-AcLys	anti-AcOrn	AAPA	RF or anti-CCP	RF or anti-CCP or anti-AcLys	RF or anti-CCP or anti-AcOrn	RF or anti-CCP or AAPA
**Sensitivity**							
All^a^	21.1	36.1	39.7	75.7	77.1	77.9	78.2
Anti-CCP-	5.6	8.4	9.6	27.9	32.6	34.6	35.4
Anti-CCP-/RF-	6.3	9.3	10.2	Na^b^	6.3^c^	9.3^c^	10.2^c^
**Specificity**							
All	94.2	91.5	90.6	79.5	75.8	74.6	74.1
Anti-CCP-	95.1	93.4	92.6	87.0	82.9	81.7	81.1
Anti-CCP-/RF-	95.3	93.9	93.1	na	95.3^c^	93.9 ^c^	93.1^c^

The antibodies were considered in isolation and combinations using the OR operator.

^a^Sensitivity and specificity were evaluated in all EA patients and the specified subsets.

^b^*na* not applicable.

^c^Values already reported on the left columns but repeated for easy comparison.

### Improved serological classification based on antibody concordance

The relations of RF, anti-CCP and anti-AcOrn antibodies presented above (Fig. 1A,B) suggested their concordance could be useful for RA classification. This hypothesis was further supported by the discrimination afforded with the number of positive antibodies relative to the serological component of the 2010 ACR/EULAR criteria (Supplementary Table S5 online). Therefore, we directly compared the serological component of the 2010 criteria with the antibody concordance in separate logistic regression models (Table 3). In the separate logistic regressions, the highest OR (48.7) corresponded to the concordant presence of the three antibodies (RF, anti-CCP and anti-AcOrn antibodies). It was followed by the concordant presence of two antibodies (OR 28.5) and at a similar level the high levels of RF or anti-CCP antibodies (OR 25.9). The same order was observed within the logistic model combining the two serological criteria (OR 11.4, 7.9 and 4.4, respectively, Table 3). This hierarchy indicates a higher weight of the concordance that could lead to better classification than with the 2010 serological component. The improvement was demonstrated with the comparison of the overall performance of the models, which was done with the R^2^ and AIC parameters. The R^2^ is better when it is larger, as it was in the concordance model (0.451) compared with the 2010 serological component (0.422). In contrast, the AIC is better when is lower, as in the concordance model (1037) relative to the 2010 serological criteria (1067). This latter difference is considered discriminant (> 10). A further improvement in performance was obtained with the combined model (R^2^ = 45.6%, AIC = 1027). In contrast, none of the models based on the concordance with other antibodies, anti-AcLys (R^2^ = 44.6%, AIC = 1038) or anti-CarP antibodies (R^2^ = 44.2%, AIC = 1048), was better than the including anti-AcOrn.

**Table 3:** Comparison of logistic regression models for RA classification based on the serological 2010 criteria, the antibody concordance, and their combination.

Stratum	2010 ACR/EULAR^a^	Ccdc. anti-AcOrn	2010 + Ccdc. anti-AcOrn
	OR (95% CI)^b^	OR (95% CI)	OR (95% CI)
3	25.9 (17.9–37.6)	–	4.4 (1.9–10.4)
2	2.5 (1.6–3.8)	–	1.2 (0.5–2.6)^c^
3Ab	–	48.7 (25.5–93.0)	11.4 (4.0–32.9)
2Ab	–	28.5 (17.1–47.4)	7.9 (3.0–20.4)
1Ab	–	2.7 (1.9–3.9)	1.6 (0.8–3.2)^c^

^a^The serological component of the 2010 ACR/EULAR RA classification criteria, the concordance (Ccdc.) of RF, anti-CCP and anti-AcOrn autoantibodies, and their combination (2010 + Ccdc.)

^b^OR and their 95% confidence intervals.

^c^This stratum did not contribute significantly to RA classification.

### Improved RA classification with the anti-AcOrn antibodies

The final test to ascertain the classification consists in combining the serological models with the non-serological components of the 2010 ACR/EULAR criteria (Table 4). This analysis was only possible in the PEARL cohort (537 EA patients) because of data availability. The results showed an improvement in specificity and accuracy with the antibody concordance of RF, anti-CCP and anti-AcOrn antibodies. In more detail, the specificity was significantly larger than with the 2010 criteria (77.6 *vs.* 72.6%, p = 0.003). This increase was accompanied by a numerically larger sensitivity (80.2 *vs.* 78.8%, p = 0.25) and a significantly improved accuracy (79.0 *vs.* 75.8%, p = 0.002). This improvement was not attributable to the specific scoring that was used as it was replicated with the logistic regression model without predefined scores (Table 4; specificities: 79.2 *vs.* 73.8%, p = 9.5 × 10^–6^; accuracies: 80.0 *vs.* 77.1%, p = 0.007). Another notable result of these analyses was that the classification incorporating a combination of the serological scores (Ccdc. anti-AcOrn + 2010 in Table 4) was not better than the based only on antibody concordance (Ccdc. anti-AcOrn in Table 4).

**Table 4:** Performance of the classification criteria for RA with different serological components.

Serological component^a^	Scores^b^			Logistic regression^c^	
	Specificity	Sensitivity	Accuracy	Specificity	Accuracy
2010 ACR/EULAR	72.6	78.8	75.8	73.8	77.1
Ccdc. anti-AcOrn	77.6	80.2	79.0	79.2	80.0
Ccdc. anti-AcOrn + 2010	77.6	81.3	79.5	80.3	80.6
Ccdc. anti-CarP	75.3	78.8	77.1	75.7	77.8
Ccdc. anti-CarP + 2010	76.4	80.6	78.8	79.5	80.0

^a^2010 ACR/EULAR = RF or anti-CCP antibodies as in the 2010 ACR/EULAR classification criteria; Ccdc. *anti-AcOrn* concordant for RF, anti-CCP and anti-AcOrn antibodies; Ccdc. *anti-CarP* concordant for RF, anti-CCP and anti-CarP antibodies.

^b^Scores were as in the 2010 ACR/EULAR criteria; as in Regueiro et al.^16^ depending on the number of concordant antibodies: 5 for 3, 3 for 2, and 1 for 1 antibodies; or combining the two criteria as: 5 for 3 or 2 antibodies with high levels, 3 for 3 or 2 antibodies with low levels, and 1 for 1 antibody irrespective of the levels.

^c^Classification was done with logistic regression including each component: joint symptoms, serology, symptom duration, and acute-phase reactants with the cut-off adjusted to obtain 80% sensitivity. This analysis is slightly different from the reported in Regueiro et al*.* where the three non-serological factors were grouped.

The improvement in RA classification with the anti-AcOrn antibodies was significantly larger than the obtained with the anti-CarP antibodies, which we had recently reported^16^, and that is shown here for direct comparison (Table 4; scores specificity: 77.6 *vs.* 75.3%, p = 0.03, and accuracy: 79.0 *vs.* 77.1%, p = 0.01; logistic regression specificity: 79.2 *vs.* 75.7%, p = 0.002, and accuracy: 80.0 *vs.* 77.8%, p = 0.01).

## Discussion

We have identified the anti-AcOrn as RA autoantibodies that in our patients improved the specificity and accuracy of the RA classification and freed it from the reliance on antibody levels. The significant improvement was obtained with one of the two explored approaches: the use of a scoring system based on the antibody concordance. In this approach, the anti-AcOrn concordance with RF and anti-CCP antibodies was superior to that of the anti-AcLys and anti-CarP antibodies. The improvements over the 2010 ACR/EULAR criteria were significant in specificity and accuracy, and not significant in sensitivity. These improvements should stimulate replication and further analysis. Only after replication and expert consensus, a recommendation for changes in the RA classification could be envisaged. The other explored approach, aimed to use the anti-AcOrn as a biomarker for the RF and anti-CCP “seronegative” patients, did not result in meaningful improvements.

Other reports have already signalled the anti-AcOrn antibodies outperform several other AAPA as RA biomarkers although they did not explore RA classification^18–22^. The combination of anti-AcOrn with anti-AcLys did not result in significant improvements in sensitivity, probably because of the high concordance between them. In this regard, it is worth mentioning the AAPA sensitivity for the “seronegative” RA patients was much lower than the observed in two previous reports^20,21^. These two reports are only available as meeting abstracts, a circumstance that limits the search for possible causes of the discrepant results. In contrast, our results are in line with the 13.2% anti-CCP negative patients that were positive for the anti-AcLys antibodies in the unique additional article with this information^18^.

The low sensitivity in the RF and anti-CCP “seronegative” patients indicates the AAPA will not be of utility to improve the classification of this subset of patients^7, 8,11–13^. Unfortunately, this seems to be the common outcome with the autoantibodies given their concordant presence in the RA patients^16–18^. Even, the concordance may reflect shared underlying pathways, as suggested by the recent work indicating that smoking predisposes primarily to RA with multiple autoantibodies^37–39^.

Independently of its causes, the concordance of antibodies is a defining characteristic of RA patients^16–18,24,27–30^, which could be exploited to improve RA classification in another way^14,15^. The improvement exploits the better specificity of the concordant presence of two or three antibodies relative to any of them in isolation. An observation that was evident in the 12 studies compiled in Verheul et al.^24^. Therefore, we have proposed a scoring system that gives a higher weight to the serologic component than the 2010 ACR/EULAR criteria (5 points for the top serological score of a total of 6 needed)^14^. This approach has led to improvements in the classification with the anti-CarP antibodies. Improvements that were independently replicated in the Leiden EA cohort^15^. Here, a similar pattern of concordance was found with the anti-AcOrn antibodies in place of the anti-CarP antibodies. This concordance criteria with anti-AcOrn led to higher specificity and accuracy than the 2010 ACR/EULAR criteria and higher also than the obtained with the anti-CarP antibodies. The improvements obtained with the anti-AcOrn concordance scores over the 2010 ACR/EULAR criteria were of significant magnitude (specificity 5% and accuracy 4.2%). The relevance of the improvement is revealed considering that classification criteria are a tool to identify homogeneous groups of patients for research^2–5^. An objective that calls for the highest possible specificity to permit replication, comparison and transference of the research results. Our results suggest it will be possible to retain the increased sensitivity of the 2010 criteria for early RA without paying the price of decreased specificity relative to the 1987 criteria (a loss quantified at 4%)^5,6^.

The independence form antibody levels is an advantage of the anti-AcOrn over the anti-CarP antibodies whose concordance scores need to be combined with scores based on the RF and anti-CCP levels to reach similar specificity and accuracy. The independence from antibody levels is also an advantage over the 2010 ACR/EULAR criteria and another previously explored modification on the criteria^10^. An important component of the latter was requiring high levels of RF to increase specificity. However, high levels of RF were less reproducible than RF positivity between the three analysed cohorts leading the authors to propose dropping RF levels from the classification criteria^10^. The same report showed that the distinction in RF levels added little to classification. This could be explained by the correlation between high RF levels and anti-CCP presence observed in our patients (Supplementary Table S6) and in other sets of RA patients^38–40^. Therefore, the lack of reliance on the RF and anti-CCP levels will permit increasing reproducibility of the RA classification based on anti-AcOrn concordance without losing accuracy.

Another aspect that could affect the reproducibility of the findings pertains to differences in the patient populations. In this respect, the frequency of smokers was lower in our patients than in some other European EA cohorts^37–39^. However, we did not find that smoking alters the association of antibody concordance with the RA classification. In effect, the OR for RA in the concordant patients for 3, or 2 antibodies (RF, anti-CCP or anti-AcOrn) were very similar in ever smokers and the global set of patients (46.8 *vs.* 48.7, for 3 antibodies and 30.3 *vs.* 28.3 for 2 antibodies, respectively).

It is important to highlight that our results do not imply the anti-AcOrn antibodies are involved in the RA pathogenesis. This is a question that should be solved with other types of experiments. Also, additional experiments will be needed to identify the natural antigen recognized by the anti-AcOrn antibodies because the peptide used in our assay is artificial in several respects^18,19^. This field is at a stage that could be similar to the experienced by the anti-citrullinated protein antibodies some decades ago^41,42^. They were first detected as antibodies against cutaneous proteins, keratin or epidermal filaggrin^43,44^. Only after years of study, it was discovered that these antibodies were targeting citrullinated proteins that are present in the inflamed joints as vimentin, fibrinogen, enolase or histones^41,42^. It is encouraging that the first natural acetylated protein autoantigens, acetylated histones, have already been identified as RA autoantigens with a possible role in pathogenesis via antibody-NETosis interactions^45^. Whereas these questions are clarified, the anti-AcOrn antibodies can be used as biomarkers because they reveal a fraction of the spectrum of antibodies not uncovered with the other autoantibodies studied here. This extreme was evidenced in the logistic regression analysis including RF, anti-CCP and anti-CarP antibodies in addition to the anti-AcOrn antibodies. These same arguments show that the improvements we have found are not undermined by the recent studies demonstrating various levels of cross-reactivity between RA autoantibodies^46,47^.

A limitation of this study is the variation between the two lots of the AAPA assays we used that required normalization and reestablishment of the threshold for positivity. We do not know if this problem was punctual or has happened with additional reagent lots. In favour of the punctual problem is the use of the AAPA assays by other researchers without reporting any technical limitation^18–23^. It should be noted that caution and appropriate controls are always needed with this type of research assays that are not commercially available, nor approved for clinical use. Also, replication of the findings on other sample collections is required to be confident in the results. This need for prudence applies particularly to the ranking of the anti-AcOrn antibodies as more informative than the anti-CarP antibodies because the differences in performance were small or non-significant. Also, it will be important to analyse EA cohorts including patients with any joint swelling, in place of the two swollen joints required in our EA cohorts. Another limitation is the possible error in the assessment of the value of the AAPA introduced by the presence of RF in the 1987 RA classification criteria that we used after 2-years of follow-up. The weight of RF in that criteria is lower than in the 2010 criteria^4^, but still, it was unclear if it was biasing the results. To assess this possibility, we compared the anti-AcOrn performance in the anti-CCP^−^ and the RF^−^ patients. A significant bias due to the presence of RF in the 1987 criteria should result in increased sensitivity for the anti-CCP^−^ patients. Reassuringly, the contrary result was found: the sensitivity for RA patients was 8.4% and 17.4% for the anti-CCP^−^ and RF^−^ patients, respectively. Finally, implementation of the anti-AcOrn antibodies will require an additional laboratory test, but the added cost and labour will not be considered too cumbersome in the research setting that is the primary place of classification criteria.

In summary, the anti-AcOrn antibodies showed the best potential for improving the accuracy of the current RA classification among the analysed antibodies. This potential was demonstrated with a scoring system based on antibody concordance. The improvement affected specificity and accuracy and made redundant the stratification according to antibody concentrations. Regarding sensitivity, our analysis did not replicate the reported high sensitivity of the AAPA in the “seronegative” RA patients. In consequence, they were not as useful for improving the sensitivity of RA classification as suggested in previous reports. Although encouraging, our results should be taken with prudence until confirmed in other cohorts, particularly concerning the relative benefits achievable with anti-AcOrn and anti-CarP antibodies.

## Supplementary information

Supplementary Tables.

## Data Availability

The datasets generated during the current study are available from the corresponding author on reasonable request.
