# Supplementary material for: Improved classification of rheumatoid arthritis with a score including anti-acetylated ornithine antibodies
Source: Sci Rep. 2020 Nov 6;10:19263. doi: 10.1038/s41598-020-73919-y (PMC7648756; doi:10.1038/s41598-020-73919-y)
Supplement: Supplementary file 1 — Supplementary Tables. [file 41598_2020_73919_MOESM1_ESM.docx]

Supplementary Information

**Improved Classification of Rheumatoid Arthritis with a Score Including Anti-Acetylated Ornithine Antibodies**

Lorena Rodriguez-Martinez ^1^, Holger Bang ^2^, Cristina Regueiro ^1^, Laura Nuño ^3^, Ana Triguero-Martinez ^4^, Diana Peiteado ^3^, Ana M. Ortiz ^4^, Alejandro Villalba ^3^, Ana Martinez-Feito ^3^, Alejandro Balsa ^3^, Isidoro Gonzalez-Alvaro ^4^, Antonio Gonzalez ^1^

^1^ Experimental and Observational Rheumatology and Rheumatology Unit. Instituto de Investigacion Sanitaria - Hospital Clínico Universitario de Santiago (IDIS), Travesia Choupana, sn. 15706-Santiago de Compostela, Spain.

^2^ Orgentec Diagnostika GmbH, Carl-Zeiss-Straße 49-51, 55129-Mainz, Germany.

^3^ Rheumatology Department. Instituto de Investigación Hospital Universitario La Paz (IDIPAZ), Paseo de la Castellana, 261. 28046- Madrid, Spain.

^4^ Rheumatology Department. Hospital Universitario de la Princesa, Instituto de Investigación Sanitaria la Princesa (IIS-lP), Calle de Diego de León, 62. 28006-Madrid, Spain.

**Supplementary Table S1.** Logistic regression analysis of the association of the AAPA antibodies with RA.

| Adjusted for ^a^ | AAPA | OR | 95% CI | p |
| --- | --- | --- | --- | --- |
| no antibodies |  |  |  |  |
|  | anti-AcLys | 4.4 | 2.9-6.7 | 7.0 x10^-12^ |
|  | anti-AcOrn | 6.5 | 4.5-9.3 | 2.3 x10^-24^ |
|  | AAPA | 6.8 | 4.8-9.6 | 3.8 x10^-27^ |
| anti-CCP/RF |  |  |  |  |
|  | anti-AcLys | 1.4 | 0.8-2.5 | 0.2 |
|  | anti-AcOrn | 1.7 | 1.1-2.8 | 2.1 x10^-02^ |
|  | AAPA | 1.8 | 1.1-2.8 | 1.2 x10^-02^ |
| anti-CCP/RF/anti-CarP |  |  |  |  |
|  | anti-AcLys | 1.4 | 0.8-2.4 | 0.3 |
|  | anti-AcOrn | 1.6 | 1.0-2.6 | 4.5 x10^-02^ |
|  | AAPA | 1.7 | 1.1-2.7 | 2.5 x10^-02^ |

^a^ All logistic regression models included age, sex and cohort as additional variables.

**Supplementary Table S2:** Association of the level-stratified ^a^ anti-acetylated peptide antibodies with the RA classification.

| Comparison | OR ^b^ | 95% CI | p |
| --- | --- | --- | --- |
| anti-CCP or RF |  |  |  |
| low *vs.* negative | 2.2 | 1.4-3.4 | 4.6 x10^-04^ |
| high *vs.* negative | 30.4 | 20.5-45.2 | 1.5 x10^-64^ |
| high *vs.* low | 12.5 | 7.3-21.3 | 1.4 x10^-20^ |
| anti-AcLys |  |  |  |
| low *vs.* negative | 3.4 | 1.9-6.0 | 3.8 x10^-05^ |
| high *vs.* negative | 5.7 | 3.1-10.4 | 1.2 x10^-08^ |
| high *vs.* low | 2.0 | 0.9-4.9 | 0.1 |
| anti-AcOrn |  |  |  |
| low *vs.* negative | 7.1 | 3.9-13.0 | 1.9 x10^-10^ |
| high *vs.* negative | 6.3 | 4.1-9.6 | 2.0 x10^-17^ |
| high *vs.* low | 0.9 | 0.4-1.8 | 0.7 |

^a^ The levels of the antibody positive patients were divided in high and low relative to 3 times the cut-off of the positive ones.

^b^ Logistic regression analysis including age, sex and cohort as additional variables.

**Supplementary Table S3:** Parameters assessing the diagnostic value of the antibodies and their combinations.

| Parameter | anti-AcLys | anti-AcOrn | anti-CCP or RF | anti-CCP or RF or anti-AcLys | anti-CCP or RF or anti-AcOrn |
| --- | --- | --- | --- | --- | --- |
| PPV ^c^ |  |  |  |  |  |
| all ^b^ | 78.2 | 80.9 | 78.6 | 75.8 | 75.3 |
| anti-CCP^-^ | 29.4 | 31.9 | 44.2 | 41.1 | 41.1 |
| anti-CCP^-^ & RF^-^ | 28.6 | 31.6 | na | 28.6^a^ | 31.6^a^ |
| NPV |  |  |  |  |  |
| all | 54.8 | 59.0 | 76.6 | 77.1 | 77.2 |
| anti-CCP^-^ | 73.3 | 73.5 | 76.6 | 77.1 | 77.2 |
| anti-CCP^-^ & RF^-^ | 77.1 | 77.2 | na | 77.1^a^ | 77.2^a^ |
| LR+ |  |  |  |  |  |
| all | 3.6 | 4.3 | 3.7 | 3.2 | 3.1 |
| anti-CCP^-^ | 1.1 | 1.3 | 2.2 | 1.9 | 1.9 |
| anti-CCP^-^ & RF^-^ | 1.3 | 1.5 | na | 1.3^a^ | 1.5^a^ |
| LR- |  |  |  |  |  |
| all | 0.8 | 0.7 | 0.3 | 0.3 | 0.3 |
| anti-CCP^-^ | 1.0 | 1.0 | 0.8 | 0.8 | 0.8 |
| anti-CCP^-^ & RF^-^ | 1.0 | 1.0 | na | 1.0^a^ | 1.0^a^ |
| AUC |  |  |  |  |  |
| all | 0.58 | 0.64 | 0.78 | 0.77 | 0.76 |
| anti-CCP^-^ | 0.50 | 0.51 | 0.58 | 0.58 | 0.58 |
| anti-CCP^-^ & RF^-^ | 0.51 | 0.52 | na | 0.51^a^ | 0.52^a^ |

^a^ Values already presented in the table to the right, but duplicated to facilitate comparison. na = not applicable.

^b^ All refers to all EA patients, the other rows refer to the indicated subset of patients.

^c^ PPV = positive predictive value; NPV = negative predictive value; LR + = likelihood ratio of a positive finding; LR − = likelihood ratio of a negative finding; AUC = area under the Receiver Operating Curve (ROC); na = not applicable.

**Supplementary Table S4:** Sensitivity and specificity of the AAPA in combinations with the RF and anti-CCP antibodies using the “AND” operator for the RA classification of the EA patients.

|  | RF or anti-CCP | (RF or anti-CCP) and anti-AcLys | (RF or anti-CCP) and anti-AcOrn | (RF or anti-CCP) and AAPA |
| --- | --- | --- | --- | --- |
| Sensitivity |  |  |  |  |
| all ^a^ | 75.7 | 19.6 | 33.8 | 37.2 |
| anti-CCP- | 27.9 | 1.1 | 1.7 | 2.2 |
| Specificity |  |  |  |  |
| all | 79.5 | 97.9 | 96.4 | 96.1 |
| anti-CCP- | 87.0 | 99.2 | 98.8 | 98.6 |

^a^ Sensitivity and specificity were evaluated in all EA patients and the specified subsets.

**Supplementary Table S5:** Contingency table of the EA patients according to serological scores of the 2010 classification criteria and the number of concordant positive autoantibodies (RF, anti-CCP and anti-AcOrn).

|  |  | Antibody concordance | |  |  |
| --- | --- | --- | --- | --- | --- |
|  | scores | 0Ab | 1Ab | 2Ab | 3Ab |
| 2010 serological criteria | 3 | 0 (0)^a^ | 57 (14.5) | 169 (43.1) | 166 (42.3) |
|  | 2 | 0 (0) | 90 (80.4) | 19 (17.0) | 3 (2.7) |
|  | 0 | 514 (93.1) | 38 (6.9) | 0 (0) | 0 (0) |

^a^ Each cell of the table contains the number of EA patients and, between brackets, the percentage of the row total they represent.

**Supplementary Table S6:** Correlation between the levels of RF and the anti-CCP status among the patients with RA. The threshold between low and high RF was defined according to the 2010 ACR/EULAR classification criteria at 3 x the upper level of normal.

| RF level | Anti-CCP  status | n |
| --- | --- | --- |
| neg | neg | 129 |
| low | neg | 30 |
| high | neg | 20 |
| neg | pos | 43 |
| low | pos | 89 |
| high | pos | 219 |
| p |  | 6.3 x10^-45^ |
